# Semantic Annotation of Mutable Data

**DOI:** 10.1371/journal.pone.0076093

**Published:** 2013-11-04

**Authors:** Robert A. Morris, Lei Dou, James Hanken, Maureen Kelly, David B. Lowery, Bertram Ludäscher, James A. Macklin, Paul J. Morris

**Affiliations:** 1 Harvard University Herbaria, Cambridge, Massachusetts, United States of America; 2 Computer Science Department, University of Massachusetts, Boston, Massachusetts, United States of America; 3 UC Davis Genome Center, University of California, Davis, California, United States of America; 4 Museum of Comparative Zoology, Harvard University, Cambridge, Massachusetts, United States of America; 5 Agriculture and Agri-Food Canada, Ottawa, Ontario, Canada; University of Vermont, United States of America

## Abstract

Electronic annotation of scientific data is very similar to annotation of documents. Both types of annotation amplify the original object, add related knowledge to it, and dispute or support assertions in it. In each case, annotation is a framework for discourse about the original object, and, in each case, an annotation needs to clearly identify its scope and its own terminology. However, electronic annotation of data differs from annotation of documents: the content of the annotations, including expectations and supporting evidence, is more often shared among members of networks. Any consequent actions taken by the holders of the annotated data could be shared as well. But even those current annotation systems that admit data as their subject often make it difficult or impossible to annotate at fine-enough granularity to use the results in this way for data quality control. We address these kinds of issues by offering simple extensions to an existing annotation ontology and describe how the results support an interest-based distribution of annotations. We are using the result to design and deploy a platform that supports annotation services overlaid on networks of distributed data, with particular application to data quality control. Our initial instance supports a set of natural science collection metadata services. An important application is the support for data quality control and provision of missing data. A previous proof of concept demonstrated such use based on data annotations modeled with XML-Schema.

## Introduction

### Annotations of ancient documents

Annotation has a long history in scholarship, a history that can inform current implementations of electronic annotations. Dickey [1] notes that dictionaries and treatises on language among ancient Greek philosophers and rhetors are known from the fifth century BC, and in Near Eastern civilizations from a millennium earlier, and attributes the systematic commentary on specific written works to the librarians at Alexandria. Classical non-electronic marginalia included distinct concepts, particularly *scholia* (*σχόλια*, singular *scholion σχόλιον*; we follow Dickey in forming the English singular by transliteration, rather than the Latin *scholium*), *hypomnemata* (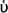
π*ο*μνηματα), and *glossia* (γλ
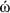
σσημα, singular *gloss*). Scholia were seen as marginal comments on the text, hypomnemata as references out to a collection of comments, and glossia as definitions, familiar to us as collections of definitions in a glossary. All three forms of annotation supplement and comment on an original text. Marginalia served a mixture of both scholarly (e.g., exegesis) and scholastic (e.g., word meanings) purposes (see, for example [2]).

Scholia are clear precursors of a number of systems of annotation throughout the history of publishing. Although various schools of linguistic scholarship had differing forms and practices for the writing of scholia, Nünlist [3] identifies several important characteristics of scholia, one of which is their composite nature. That is, a scholion has a structure; it is not simply a block of marginal text.

### Proofreading in the hot-metal type era: annotation for quality control

In the technologies immediately preceding the era of electronic publishing, the proofreading phase of publishing started with a copy editor annotating the author's manuscript with marginalia to guide the typographer. Lead slugs were cast from lines of brass molds, or, in earlier times, lines of individual metal letters were produced. These were then printed in order and the author received “galley proofs” on which to make handwritten annotations signifying errors and their expected correction. Galley proofs were thus printed with the typefaces and line breaks as planned for the final publication but not with full layout. The proof sheets had extra wide margins to support the annotations, which often used small, specialized notation (“proofreaders' marks”). Sometimes authors would also indicate a request for new material directly on the proof sheets, but additions at this point in the process were discouraged due to the potentially high cost of rearranging the intermediate rendering technology. Similar marks were also often adopted by reviewers and authors during the preparation of a manuscript (e.g., by authors in communication with a typist) and in the peer review process. For contemporaneous usage see Chapters 2 and 3 of the 13^th^ edition of The Chicago Manual of Style, especially p. 52 and p. 95 [4]. Proofreaders' marks remain documented in the current edition [5]. In a later stage of production, a similar annotation of “page proofs” addressed the full pagination details, but the cost of changes at this point was extremely high. Implicit in the application of proofreaders' marks to a manuscript or galley proofs is the expectation that the underlying document will be corrected. As such, proofreaders' marks are part of a quality control process in publication.

### Annotations of digital objects

Conceptually in the lineage of marginalia, Annotea [6] is an early standard for annotating electronic documents. Annotea identifies several key concerns of annotations: the digital object being annotated, the assertions made in the annotation, and the annotator. These structured elements can be compared with the five basic elements of a scholion identified by Nünlist, p. 8 [3]:

(i) the lemma (i.e., the verbatim quotation of the passage under discussion;…); (ii) a translation of (part of) the passage; (iii) a paraphrase of (part of) the passage; (iv) quotations (e.g., of parallel passages); (v) the commentator's own words (e.g., explanations).

Two recent efforts derived from Annotea, the Annotation Ontology (AO) project [7] and the Open Annotation Collaboration (OAC) [8], also focus on document annotation. A joint effort to reconcile AO and OAC, in which two of us (R. Morris and P. Morris) are participating, has emerged as a community project of the Worldwide Web Consortium. That effort, named the *Open Annotation Community Group* [9], has developed a community draft standard for digital annotations of digital resources called the *Open Annotation Ontology*, simply denoted OA. This paper is based on the second Community Draft Data Model [10], which expresses key concerns in a way that can ultimately lead to an ontology expressed in the Web Ontology Language (OWL), as did its predecessors AO and OAC. Those concerns include the identity of the object being annotated (the OA *Target*), the assertions made about the Target in the annotation (contained in an OA *Body*, also with an identifier), and metadata describing when the annotation was created and by whom. When the actual intended portions of the Target or Body electronic resources do not themselves have an identifier, but rather are part of an electronic resource that does, OA provides a mechanism called a *Selector* to extract those portions. We describe Selectors later, but a typical example might be the selection of a page in an e-journal article which itself has a Digital Object Identifier [11] that allows the article as a whole to be accessed.

Item (i) in the structure of scholia, the lemma, corresponds directly to a Selector that incorporates portions of a document that is being annotated in OA. As well as for selection of text, OA provides several types of Selectors applicable to a number of contemporary digital media, namely images, audio, and XML documents. In addition, communities of practice can provide their own Selectors. For data annotation, we will show how *queries* on data sets can provide Selectors, thereby providing a way for an annotator to indicate that any data returned by the given query should meet the assertions provided in the Body.

Agosti et al. [12] extensively investigate the requirements for document annotation systems. Their analysis is framed by the terminology in wide use throughout the history of document annotation (including scholia and proofreading of documents during editing). For digital objects, such as databases and the data in them, the structure of, and access to, the subject of an annotation (i.e., the OA Target) has a less static organization than do web document-like artifacts. Consequently, we prefer to characterize the differences between different data annotation system architectures along different criteria. These differences are determined by how the annotated resources are managed and accessed more than by the structure of the annotations. Consideration of those kinds of architectural differences led us to consider the impact of different ways to resolve various (appropriately) underspecified aspects of AO, and, subsequently, OA. Specifically, we have seen in production or design: (1) systems in which the annotated resource and annotation are stored and presented in the same place, analogous to scholia; (2) systems in which annotated resources are stored in various places (e.g., different database servers), copies of the data are aggregated, and the aggregated data are given a common presentation that is annotated and associated with a central annotation store (e.g., The Atlas of Living Australia's annotation mechanism in its portal for the presentation of aggregated Australian biodiversity data [13]); (3) systems in which annotations and annotated resources are stored together, but in which the annotations carry pointers to resources stored somewhere else, analogous to glossia. Examples are the images [14] associated with the Encyclopedia of Life species page for *Pinus strobus* (the eastern white pine) [15]. Links are provided to the sources of these images, and the images are annotated as either “unreviewed” or “trusted” depending on whether or not a page curator has determined them to be correctly identified; and (4) systems in which data are distributed and annotations are transported from places where data are presented to all places where related data are stored (“annotations in motion,” e.g., our own FilteredPush system [16]). Cases (2) and (3) are typical of semantic web systems, where resources are linked by pointers to related resources (and there is no concept of transport of annotations), whereas case (4) involves mixtures of semantic web and other technologies. While other models exist, this categorization has helped us differentiate the concerns of annotation from those of transport and presentation.

### Context of the present work

In this paper, we discuss enrichment of OA to better treat annotation of data. An earlier draft focused on issues we encountered with AO, but some of these have been addressed in OA. Occasionally to give perspective to our use of OA we mention AO. Our initial use of data annotations is to support quality control, including the provision of missing data for distributed data comprising specimen metadata in natural science collections. (Following common practice we refer to this as the *specimen data*, but keep in mind that the specimens are physical objects.) Our original focus was on botanical collections, which have some particularly vexing specimen data quality problems arising from the practice of botanists of collecting multiple cuttings from the same organism, gluing each to a separate piece of paper (a “herbarium sheet”) along with a label containing the specimen data, and distributing some of these sheets to their colleagues at other herbaria [17]. Over time, this practice produces diverging paper and electronic specimen data records of the same biological individual. In principle, the specimen data about the taxonomy and about the place and time of the collection should be the same for all these “botanical duplicates.” In practice, it is not, because scholarship about these specimens proceeds independently as scholars examine and annotate specimens in some collections but not others. Thus, the curation proceeds at sometimes radically different paces, often without effective communication between curators. These expected, but often missing, correlations between the data are, however, common among many kinds of specimen metadata. In practice, when a user of such data is able to discover, and perhaps offer remedies for, missing or erroneous data, the result rarely is communicated to the original data holder. Our motivation for the extensions that we proposed to OA arose from our requirement to provide this notification by *pushing* the data to all interested parties, who can then filter these notifications, regarded as annotations on the published data, to meet their scientific and curatorial workflows. In designing the FilteredPush platform, we concluded that it required us to address several missing or underspecified facets of knowledge representation in AO, how, if at all, they were addressed in OA, and, if not, what extensions we continue to believe are necessary. Much of the current work in the annotation of electronic resources, including OA, has a focus on web documents. OA has addressed some of the difficulties we faced using AO for data annotations.

Distributed annotation of distributed data leads us to three principal concerns: data elements as subjects of annotations, transport of annotations of distributed data to remote consuming data curation applications, and, as for proofreaders' marks, annotations that seek to change, not just supplement, the annotated data.

## Methods

### RDF and the OWL Web Ontology Language and its use for annotations

OWL [18] is a standardized controlled vocabulary for describing controlled vocabularies, including classifications of terms, relationships between terms, and strong datatyping. OWL is based on a less restrictive standard, the Resource Description Framework (RDF) [19], which is the major foundation of the Semantic Web [20]. Other ontology languages are in wide use in the biological sciences, most notably those expressed in the Open Biomedical Ontology (OBO) format [21], which is particularly used for molecular biology and evolutionary biology [22]–[24]. Among other things, OWL distinguishes itself from OBO by a theoretical foundation that allows for logical reasoning by machines in ways that can control the tractability of computation. However, recent efforts to characterize mapping between OBO and OWL may reduce the consequences of this [25]. The latest OWL version, OWL2 [18], provides close connection to one or another more familiar facilities for data modeling, such as relational databases. Tractability includes ensuring that machine reasoning does not fail to reach a logical conclusion and does not require resources that grow exponentially with the size of data. All that said, less expressive languages such as OBO often are suited to less demanding semantic issues such as locating data that are described by synonyms or perhaps narrower terms than those used in a search. OWL, and, more generally, the RDFS specialization of RDF, provide several major kinds of knowledge representation mechanisms to describe objects (more technically called “resources,” but we use the terms interchangeably): *Classes*, which classify objects; *Individuals*, which are members of a Class that is said to be a *type* of the individual; *Object Properties*, which relate objects to objects; and *Data Properties*, which relate objects to data such as strings, dates, and numbers. Resources are given a globally unique identifier conforming to the Uniform Resource Identifier (URI) specification [26]. Properties are often called *predicates* following the formal syntax of RDF. In practice, few if any Individuals are described *within* an OWL ontology. Rather, Individuals more often model digital or physical resources described *by* the terminology defined in the ontology. See [27] for an introduction to RDF, RDFS, OWL, and the SPARQL RDF query language.

The OA data model expresses its concepts in several principal formal constructs:

- a Class *Annotation*
- an Object Property *hasTarget*, whose value is the subject of an annotation, colloquially called the *Target*
- a Class *Selector* and a predicate *hasSelector*, which specify a part of the annotation Target to which the annotation applies, if less than the whole Target
- an Object Property *hasBody*, whose value contains the assertions about the subject of the annotation; the object of hasBody is colloquially called the *Body*
- a small collection of provenance properties that establish who created the Annotation as well as when and what software may have generated it
- a Class *Motivation* and a predicate *motivatedBy*, which assist annotation producers to express the reasons for their construction of the annotation.

## Results

### Extensions to OA

The original focus of AO, OAC, and, subsequently, OA, was the annotation of documents with emphasis particularly on human consumption, though the OA model supports signifying that some content of an annotation may be aimed at machine processing. In some cases, we and others argued successfully for treatment of concepts missing in earlier drafts of OA (discussed below where relevant). Of particular note is a relaxation of the restriction in an earlier draft that there is at most one Body. In other cases we find that we need some concepts not yet agreed to as germane to annotation of arbitrary resources. For convenience and clarity, we identify the proposed new terms with the prefix “oad:” as an abbreviation for its formal namespace. “rdf:” designates terms from the RDF vocabulary itself, and “cnt:” refers to an impending W3C Recommendation “Representing Content in RDF 1.0” [28] (hereafter “Content in RDF,” CNT). The “oa:” prefix denotes terms currently in the OA proposed specification. The Supporting Information includes complete lists of the namespace abbreviations [Table S1] and acronyms [Table S2] used herein.

To OA we add two primary classes in an ontology [Ontology S1] we denote OAD (Open Annotation for Data): *oad:Expectation* and *oad:Evidence*, as well as some lower level classes, which we discuss in the section **“Selectors and queries”** below. We have introduced Object Properties *oad:hasExpectation* and *oad:hasEvidence* to support the use of the corresponding classes.

In the FilteredPush prototype we implemented annotations using an XML-Schema, which provided for objects that are sometimes opaque to the software transporting the annotations through a network and sometimes not. Long human-generated strings are a typical example of opacity. For our subsequent use of AO we provided a string-valued Data Property “*asText.*” OA adopts the Content in RDF vocabulary for this same purpose. That vocabulary also allows completely opaque objects to be transported in annotations in ways that only consuming applications can understand. Examples of this are embedded images and encrypted text, which we discuss at greater length in the section **“Opaque objects”** below.

All figures, tables, and examples refer to OA and OAD, as well as to a purpose-built OWL ontology [Ontology S2] that we denote the *DarwinCore FilteredPush Model*, with prefix “dwcFP:” It is a representation of the DarwinCore (“DwC”) standard [29] that describes, among other things, natural science specimen records. Darwin Core is promulgated in a number of forms by the Biodiversity Informatics Standards (TDWG) group [30].

### Motivation for the extensions in support of mutable data

We have been motivated to extend OA to a case somewhat distinct from the purpose of classical scholia, but similar to proofreaders' marks—the annotation of mutable scientific data. We have been participating in the OA Community Group to help ensure that this use case is expressible as that proposal goes forward. In most of science, data are usually seen as immutable, perhaps transformed into different representations, but fundamentally retained as the actual measured values from some observation. In some domains, however, such as the data associated with natural science collections, or vouchered observations of occurrences of organisms in biodiversity science, some elements of data sets are expected to change over time. For example, the scientific names applied to an organism may change over time. Moreover, even intentionally immutable data are subject to inadvertent change as they move around the Internet passing through a series of servers and software. Also, scientific conclusions are often based on derived data, such as might be provided by statistical or other mathematical analyses. Those analyses are sometimes arguably inappropriate even if nominally correct. On scientific grounds, such data deserve annotation expressing a scientific opinion about their fitness for the purpose to which they may have been put.

The annotation of data sets that can be expected to change differs from the classical annotation of paper documents and their modern digital counterparts in one very important way: the author of a scholion would not expect the scholion to induce a change to the text being annotated. An author of an annotation on an electronic data set may very well expect her annotation to cause the data curator to change the actual data set, with the annotation perhaps being retained as provenance for the change.

In natural science collections, the vast majority of data are specimen metadata—dark data existing mainly as several hundred years of paper records documenting an estimated three billion specimens of non-microbial biota alone [31]. A small portion of these data, perhaps at most 3% [32], has been transcribed into digital form from paper records (and a much smaller portion was born digital). The majority of these digital records are maintained in relational databases. Transcription errors are known to exist in some of these data, so digital datasets that document what organisms occurred where and when are clearly subject to correction. Additionally, specimens have often been separated from rich sources of information about when and where they were collected. For example, field notes from a collector may reside in the archives of one institution, while that collector's specimens are distributed amongst many institutions, each with only very limited paper records pertaining to the specimen. Thus, linking a transcribed minimal set of information to a richer source of information can augment the data available about when and where a particular specimen was collected. There is also a deeper process of change intrinsic to the data. Specimens and observations of organisms are subject to the nature of taxonomic science: opinions about the scope of species can change over time, so that the identifications applied to individual specimens are also expected to change over time as experts view the specimens and change their identifications to reflect changing taxonomies. There are thus multiple reasons for the curators of a scientific dataset in the domain of biodiversity science or in natural science collections to accept and, in fact, welcome changes to their data sets over time.

### Expectations for mutable data

The changes that a curator of a biodiversity data set might accept take at least two forms: a correction to the data set (“You've misspelled the country name, here's the correct spelling”), and the addition of new information (“Here is a new species identification for this specimen”). Both of these kinds of changes are expressions by an annotator, but the ability of the data holder to act upon them clearly depends both on the data holder's scientific and local policy decisions and also upon the capability of the data storage system holding the data that is being annotated. For example, a small number of older specimen data management systems have data models that are incapable of storing the history of taxonomic identifications assigned to a specimen. Similarly, most specimen data management systems do not allow multiple georeferences to be applied to the same specimen, a situation that arises when attempting to deduce cartographic boundaries from specimen data containing only place names and descriptions. These cases clearly illustrate that an annotation may express an unfulfillable expectation about how the receiving data curator should act. Software or human action at the data holder's site can be guided by consideration of the annotator's expectation of the outcome, along with sufficient information for the recipients to assess the trustworthiness of the recommendation, but they may be unable to act upon it for technical or policy reasons. In the section **“Annotation conversations,”** we discuss how response annotations describing whether and why the expectation was or wasn't met can provide a knowledge base of the history of changes, even when the primary database cannot. A classical scholion carried an expectation that it supplemented an existing text, i.e., that it expressed a translation or commentary on that text, not an expectation that the original text would be changed in response to the scholar's assertions. A proofreader's mark, in contrast, carried an expectation that the typesetting would be changed.

From these practices, we introduced an extension to AO and OA that we term the *Expectation* class. Expectation objects carry the annotator's expectation of how a recipient of the annotation will act upon the annotated object. We proposed five distinct expectation types: three modeled by most data storage architectures and two from our experiences with biodiversity data. These are: *Update, Insert, Delete, Group*, and *Solve with More Data*. We introduced corresponding subclasses of Expectation: *Expectation_Update* carries the annotator's expectation that the consumer of an annotation will correct her data. For example, an assertion that a country name is misspelled, or an assertion denoted by a proofreader's mark, carries an *Expectation_Update*. *Expectation_Insert* denotes the annotator's expectation that the consumer of an annotation will supplement her data set with new information. A new identification of a biological specimen and some kinds of scholarly annotations (as opposed to most proofreaders' marks) each carry an *Expectation_Insert*. For example, a marginal annotation of a publication might assert that an internal argument is incomplete without the addition of something described in the Body. *Expectation_Delete* rounds out this set of standard database operations.

*Expectation_Group* derives from our experience in the development of the FilteredPush prototype. Many desired operations on biodiversity data can be thought of as set operations (e.g., “add a member to a set,” or “remove a member from a set”). For example, botanists' practice of distributing multiple botanical sheets results in herbarium collections that often contain many sheets that are parts of sets of duplicate specimens, each set representing a single biological individual, comprising a set of specimens that spans a number of collections. One of the goals of FilteredPush is the identification and grouping of these sets of duplicate specimens. *Expectation_Group* is possibly only one of several expectations needed to deal with such sets of data, but it corresponds well to important familiar operations on scientific data, e.g., clustering.

Finally, *Expectation_Solve_With_More_Data* is a case triggered by our analysis of an email message sent to one of us (Hanken) in his role as Curator of Herpetology of the Harvard Museum of Comparative Zoology (MCZ). This email asserted that three particular MCZ specimens, which were identified as a particular species of salamander and reported as having been collected in the Dominican Republic, were not correctly identified. The sender wrote that this identification could not be correct, because salamanders do not occur anywhere in the West Indies. He could not express an opinion on the correct identification without seeing the specimens, only that the data that the record attributed to the MCZ was inconsistent with the annotator's understanding of biological reality. On investigation, the error turned out to be the result of intermingling two sets of records during transcription from paper to electronic records. (The names of three salamanders from North Carolina had been given the localities for three other amphibians from the Dominican Republic.) We thus proposed *Expectation_Solve_With_More_Data* to accommodate such situations in which the annotator believes that the data as presented are incorrect, but that someone else needs to find more information in order to resolve the error. We suspect that this term is not sufficiently rich, or perhaps not sufficiently generally named, to carry all cases of an annotator's belief that there is an issue in the data that she can't solve from the presentation of the data she is observing, but we propose this subclass of Expectation to cover the general issue.

### Evidence

Because a quality control annotation typically expresses an expectation of the behavior desired of the original data provider, the annotator needs to give that provider good reasons and incentives to meet the expectation. One such way is to indicate the annotator's motivation for providing the annotation in the first place, but, in science, it is arguably even more important to indicate the evidence that supports the assertions of the Body of the annotation. In AOD, we regarded Evidence as a special case of Motivation and noted that the evidence for an assertion is knowledge without which the assertion may not even be deemed scientific knowledge. AO, and early drafts of OA, had a Motivation class that was used to classify the Annotation, and our proposed data annotation extensions to those ontologies defined *Evidence* as a subclass of *Motivation* to model the provision of evidence as a motivation. The current community draft of OA still uses motivation as a way to classify annotations, but this is now modeled with objects in the *Concept* class of SKOS [33] along with a way that communities of practice can add to or refine the twelve OA declared classifications [34]. To the current model of OA we propose to add the motivation *oad:providingEvidence* and *oad:transcribing* as SKOS Concepts. Whether or not providing evidence does stand as a motivation in some particular case (where the body of the annotation would also be expected to assert the evidence), we also now treat Evidence as a class in its own right to provide evidence for the assertions made in the Body. We will propose to add Evidence to OA as the OA Community Draft advances. We also would propose an object property *oad:hasEvidence* to associate the particular evidence to the annotation. This allows annotators to assign Evidence whether or not they wish to assert that doing so acts as a motivation.

The assertions of an oad:Evidence object are meant to provide evidence for the assertions in the Body of an oa:Annotation. These are distinct from assertions made within a selected part of the document about evidence for other assertions *in* the document. They are also distinct from assertions within the Body that the Target is evidence for some scientific hypothesis, possibly one not even in the document. The latter two cases are important for the use of OA to clarify the discourse within a scientific document. Thus, using OA alone, an annotator might argue that a certain selection of text is evidence for an argument elsewhere in the text, even if the author had not said so. We emphasize that the evidence for assertions in an annotation is distinct from an annotation that asserts that part of a document is evidence. In contrast to annotations about discourse, our introduction of Evidence is intended to help the data provider decide whether or not to meet the Expectation of an annotator.

**Figure 1:**
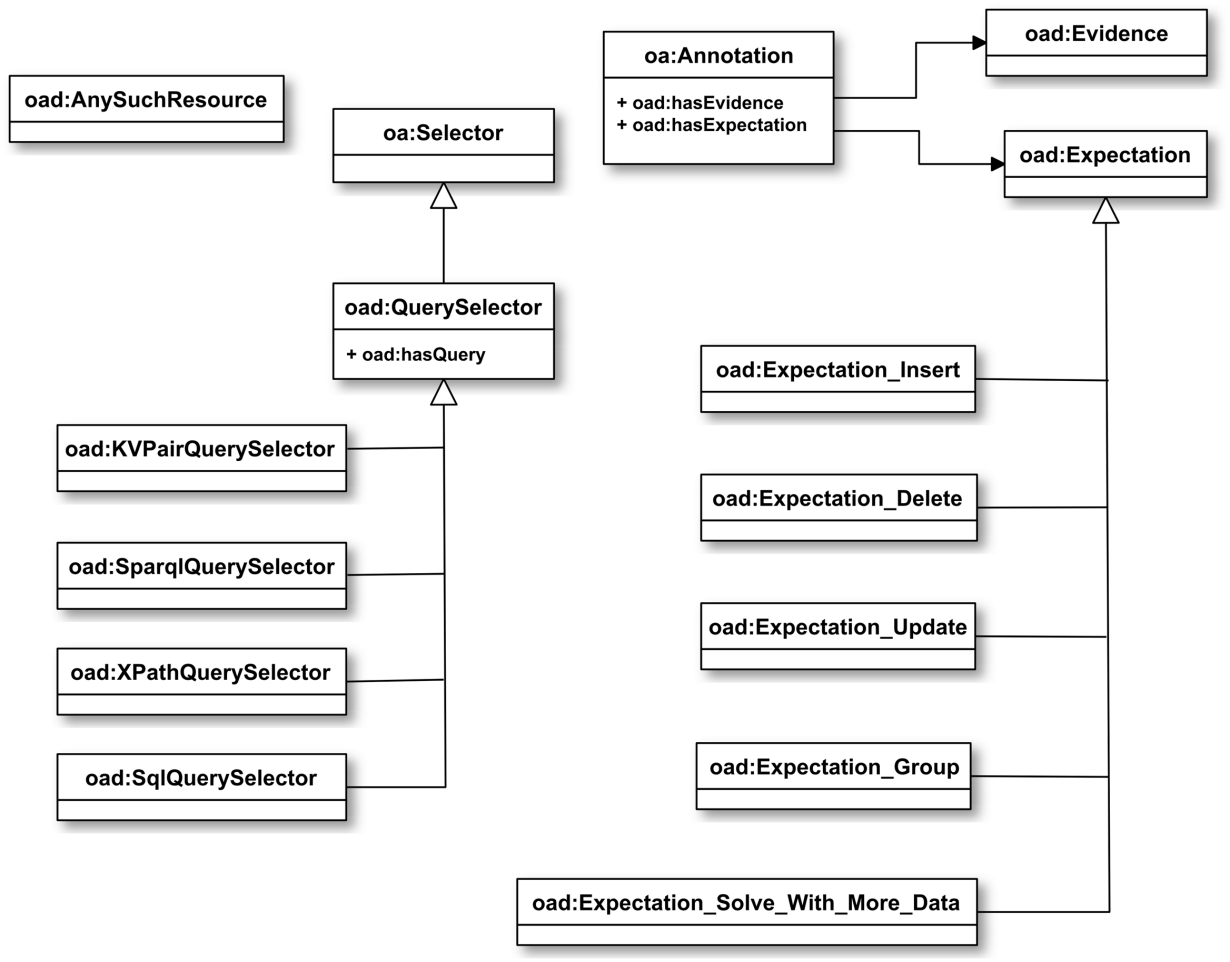
Class structure of OAD. The diagram follows standard UML class diagram conventions. White arrows point from subclass to superclass. Black arrows point at the class that is the range of the property from which the arrow originates.

[Figure 1] summarizes the OAD class structure. Some of the classes are described later in the paper.

The provision of evidence is only one kind of motivation for annotating data. Consideration of annotation dialogs, i.e., annotations upon annotations, can reveal some motivations more general than the provision of evidence. Consider the case of an annotation with an Expectation_Update. In a networked environment such as FilteredPush, this Expectation may have become moot because the data have changed since the annotation was launched into the network. Whether or not that change meets the original Expectation, the recipient may reject the annotation based on the fact that the Expectation and even the Evidence are now moot. The recipient might indicate that decision in an annotation whose Target is the original annotation. Some motivations in OA, such as oa:tagging or oa:commenting, have similar concerns to oad:Expectation_Insert, but, in general, an oa:Motivation, in expressing a reason for the creation of an annotation, might express concerns orthogonal to those of oad:Expectation—the annotator's expectation about the action of consumers on their *data*. For example, oa:tagging and oa:commenting could reflect a desire for the Body to be memorialized with the annotation, not with the data.

Finally, specimen data are not particularly hard to discover on the web, in no small measure due to the efforts of the Global Biodiversity Information Facility [35], whose data portal [36] aggregates and serves over 400 million records from over 10,000 datasets as of August 2013. (A large fraction of these are not specimen data, but rather occurrence data provided by expert observers, e.g., 97 million records from the Avian Knowledge Network [37].) Among the issues is that GBIF is known to have multiple copies of what are identified as the same record but that have some different attribute values. We conclude from this that, in this domain, more important than discovery, one of the qualifications for the utility of an annotation ontology is whether it provides users a scaffold on which to express, in the domain vocabulary, their scientific knowledge about the primary data and the data's fitness for use.

### Competency questions for annotation of mutable data

One highly useful ontology development tool is the *competency question*. Competency questions allow a designer to evaluate whether a version of an ontology is able to serve its intended purpose. (See [38]) We have developed a number of competency questions that have shaped our extensions of AO and OA to the annotation of mutable data. Some of these competency questions are phrased as general questions at the level of any annotation; others are phrased as domain specific questions. Here, we only give examples of competency questions as questions in English; a further step is often the expression of them in a query language supporting the expressivity of the ontology language. In our case that query language is SPARQL. Some examples of general applicability are:

- What evidence for this annotation would cause me to accept its recommendations into my datastore?
- What action does the annotator expect me to take on my database if I accept this annotation?
- When an ontological term that is used as evidence for annotations changes because of new data or interpretations, which annotations need to be reexamined in light of that change?
- Has the evidence that annotators have made in proposing annotations been consistent, or is there conflicting evidence being used to support the same propositions?

We next give examples of domain-specific competency questions couched in terminology common among taxonomists. For formulation of these questions into queries suitable for machine reasoning, these questions and the topics of the annotations would typically be expressed in a domain-specific controlled vocabulary.

- When asserting that a specimen in my collection should be identified as *Cornu aspersum*, is the annotator expressing a new identification based on observed characters, or correcting the nomenclature?
- What annotations about species determinations are supported by evidence that includes the presence of a morphological feature described by a now deprecated term, a term that is now understood to represent an analogous feature and has been replaced by several different terms representing non-homologous features?
- Find all the sets of descriptive characters that have been presented as evidence for any known determination of individual specimens as members of *Helix aspersa*. Are these sets congruent?

All of these competency questions are addressed by oad:Expectation and oad:Evidence, provided there is sufficient domain vocabulary to express the underlying assertions and their negations. We give some examples of SPARQL formulation of some of these in [File S1].

### An example

**Figure 2:**
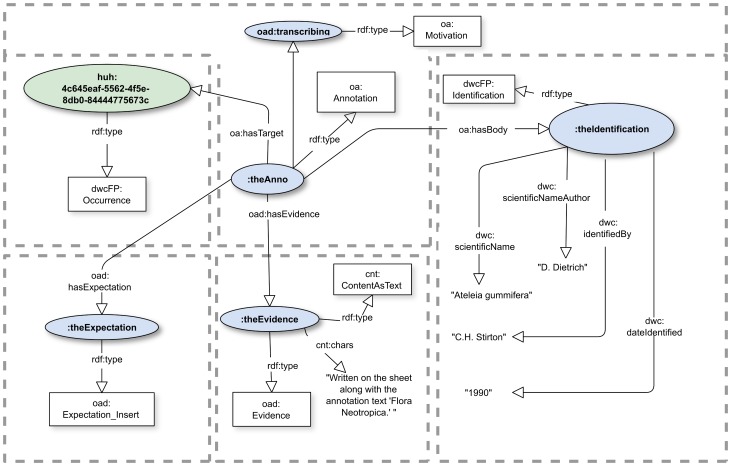
Annotation providing a taxonomic identification. Figure illustrates an abbreviated annotation providing a taxonomic identification for an occurrence record. The record is selected by reference to a lengthy identifier in the namespace of the Harvard University Herbaria (prefix “huh:”). [RDF S1] is a complete RDF representation in N3 syntax. The prefixes“oa:”, “oad:” and “dwcFP:” indicate terms respectively from the Open Annotation Ontology [61], the extension ontology we propose [Ontology S1], and a purpose built OWL ontology [Ontology S2] representation of the Darwin Core vocabulary [29].

A typical example, which illustrates an annotation suggesting a taxonomic identification of a previously unidentified specimen, is shown in [Figure 2]. For simplicity, the example omits important provenance information, such as the creator and creation time of the annotation.

In the Figure, ovals designate objects, arrows designate predicates named by their labels, and rectangles designate classes. “rdf:type” designates to which class a particular object belongs. The prefix “huh:” designates identifiers of specimen records in the Harvard University Herbaria; this particular specimen is in the herbarium of the Arnold Arboretum (A). “dwc:” prefixes the identifiers of string-valued predicates drawn directly from the Darwin Core vocabulary. We discuss dwcFP: in greater detail later. The empty prefix “:”—sometimes called the “default prefix”—does correspond to a namespace whose precise specification is often irrelevant, albeit required. The complete examples in the Supporting Information illustrate how prefixes are associated with namespaces.

The annotation depicted diagrammatically in [Figure 2] might be encoded as shown below in the RDF Turtle representation [39]. Each “sentence” corresponds to one of the dotted boxes in the Figure:
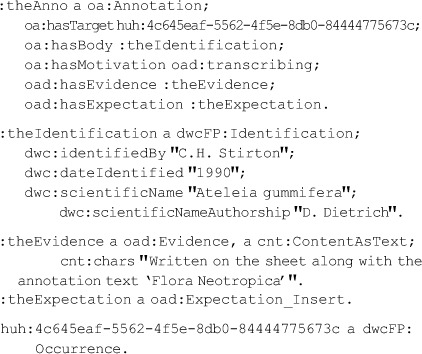



### Selectors and queries

An oa:Selector narrows the scope of the subject of an Annotation. In the context of documents, it can select the entire document or part of the document (e.g., paragraph, phrase, word). Core OA allows for the typing of a resource (e.g., target or body) as an *oa:SpecificResource*, with functionality that delineates the portion of the resource that comprises the subobject of interest, even when that portion has no identifier that references it directly, unlike the Target in the simple example above.

**Figure 3:**
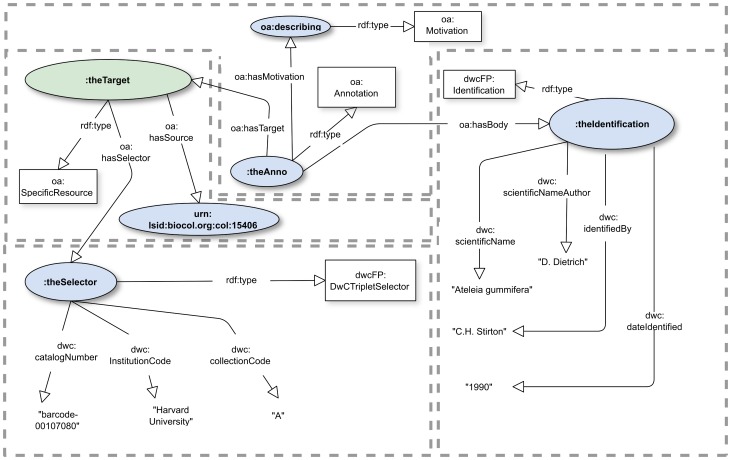
Taxonomic identification of a record identified with a set of domain terms. Note that the Selector is particular to the Darwin Core, so is part of the dwcFP ontology, not the oad ontology. A complete RDF representation is in [RDF S2].

It is common that an online dataset has a persistent URI but that individual records do not. However, such individual records are often exposed via online queries such as the key-value pairs given by RESTful web interfaces [40]. Such queries can be modeled with OA Selectors applied to Specific Resources. To this end, we exploit the fact that Specimens described by the Darwin Core are often identified by a “Darwin Core Triplet” (also called “Darwin Core Triple”) consisting of a standardized institution code, collection code, and catalog number. In OAD we provide a class *oad:KVPairSelector* meant to model extraction of data by key-value pairs. In dwcFP, we specialize this with a subclass named *dwcFP:DwCTripletSelector* to provide the Darwin Core Triplet. That Selector is in the dwcFP vocabulary, where it is defined as a subclass of oad:KVPairSelector. The model is, in part, shown in [Figure 3], which omits Evidence and Expectation to save space. Compare this with [Figure 2], which assumes the annotated record has its own URI. The entire RDF models are given in [RDF S1 and RDF S2].

### The gist of the DwCTripletSelector usage in [Figure 3] is represented by this RDF:



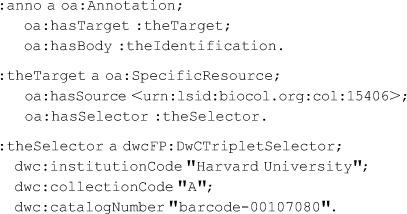
When used on a Target, the semantics of such a Selector are such that the assertions of the Body apply only to whatever (presumably in this case unique) record in the data set will produce the given triple of values. The point of providing a Selector is that there is no globally unique identifier for the record known to the annotator, but there is nevertheless a way to reference the record. In such a circumstance, it may be tempting to *construct* an identifier from the components of selector values and depend on the identifier syntax to provide for the access to the information given in the record. This is regrettably common and brings with it many thorny issues. For example, imagine that the specimen in this case became the permanent property of another institution; in all likelihood a new triplet would be issued, a new identifier provided, and, with it, the need to provide discoverable information to the effect that the two identifiers actually represent the same specimen. Page [41] addresses this issue well for Darwin Core Triplets.

Roughly, the selectors defined in core OA denote ranges in text documents or regions of interest in media objects. However, this is an oversimplification; the *oa:FragmentSelector* provides for referencing a specification, the conformance to which defines the mechanism for extracting a portion of the resource. Such specifications are expected to be “like” those of the recent W3C Recommendation for Media Fragment URIs [42]. That Recommendation is aimed at descriptions of media fragments, but the OA data model documentation argues that it is, in principle, more general. Presently, fragment selectors specified in the data model, and those that follow the goals of the Media Fragment URI Recommendation, all focus on specifying a portion of a particular serialization or representation of, in the broad sense, a document. This can be useful when the serialization *is* relevant, for example, when a referenced URI is an image from which a portion is the actual resource of interest. Example [RDF S7] shows an annotation in which two objects of Evidence are offered, one plain text and one a fragment of an image. However, by design, none of the media fragments of the Recommendation is appropriate for semantically selecting a portion of a structured data set independent of its serialization. Before turning to a model that can do so, consider the case in which the collection latitude of a collection object has been recorded as 91.3 degrees, albeit erroneously. That clearly invalid value could be the result of latitude and longitude being exchanged at some point in the data provision, but imagine that the annotator has no remedy to offer but only wishes to call attention to the problem. To model this in the Darwin Core Triplet example above, it would suffice simply to indicate the issue in the annotation Body, either with plain text or, by community agreement, with some identifier signifying a particular kind of quality control issue. In the former case, the example might contain the Body assertions:



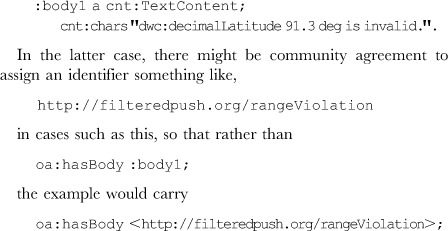
Selectors in the context of relational data sets might select the entire data set, a row within a table, a column within a table, a set of cells, or such objects within a database view. More generally, no matter the nature of the data (even if loosely structured such as in a spreadsheet, semi-structured data such as XML, data expressed as RDF, or data in a NoSQL database [43]), selectors on a dataset could select the entire dataset or the results of a query, provided only that there is a reasonable query language. On the surface it would seem that we need to know not only the URI of the Target resource but also the format of the result of applying the selector. But, in fact, there is an important use where this need not be the case. This use occurs where we mean to signify that the Body applies to all those portions of the data that meet the query, with the intention that the assertions of the Body will hold for *any* dataset, extant or in the future, and no matter how the query is applied. An example is a quality control assertion that geographic latitude data greater than 90 degrees or less than −90 degrees are always incorrect. To support this scenario, we propose the addition of several classes in the extension OAD of the OA ontology, comprising an *oad:QuerySelector* together with some particular subclasses identifying the query language. Currently, these are *oad:KVPairQuerySelector, oad:XPathQuerySelector*, *oad:SparqlQuerySelector*, and *oad:SqlQuerySelector*. Indeed, though not in OAD, the above mentioned dwcFP:DwCTripletSelector is a subclass of oad:KVPairQuerySelector. A Target that is an oa:SpecificResource with a QuerySelector will allow the specification of a portion of a *particular* dataset as the Target of an annotation. The annotation below illustrates this. It makes use of two Bodies, the first a URI for machine processing that perhaps signifies an out of range exception should be raised when applying the query. (The actual action might be left to the consuming agent.) The second Body is useful for delivery of explanations to the human interface in an annotation consuming application.



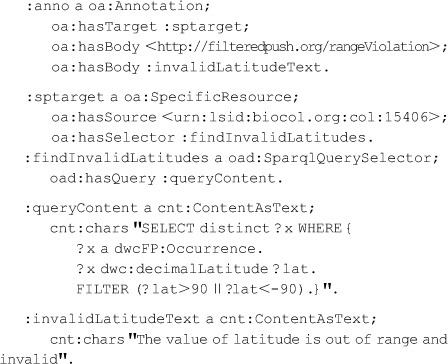
Now suppose we wish the oad:QuerySelector to apply to *any* dataset, extant or not, provided only that the query can be applied. For all practical purposes, our goal is to introduce a *variable*, something that is not modeled in RDF. One means to signal this would be to provide a target that generalizes oa:SpecificResource to include non-specific resources. However, we would rather build upon the interdependencies of SpecificResource, Source, and Selectors in current OA. Current OA requires that a specific resource must have one and only one source. We take advantage of this by declaring a special class, *oad:AnySuchResource*, with no formal semantics. AnySuchResource can be used as the source for a SpecificResource in combination with a QuerySelector. Software consuming an Annotation referencing oad:AnySuchResource is expected to process the annotation in the face of an actual resource as though that annotation were made with the URI of the actual resource. How to do so is up to the consumer. Specifying expected, albeit unenforceable, programming semantics on specific classes is seen at some points in OA itself, as in the models for aggregating multiple resources, which we discuss later. The previous example, generalized to any dwcFP:Occurrence with a dwc:decimalLatitude is the same as above except for :
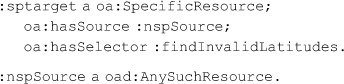



“:nspSource” is meant, in the example, to evoke that the source is a “Non-specific resource.”

We can phrase a series of general and domain specific competency questions to frame the problems that we are addressing with query selectors and oad:AnySuchResource. But recall that formalizing such questions may depend on the appropriate query language. The goal in formalizing a competency question is to ensure that our ontologies are expressive enough to provide answers to the competency questions. The oad:QuerySelectors and oad:AnySuchResource do not per se provide for queries directly so much as they provide guidance to consuming applications. Roughly, that guidance may be expressed as:

1. Determine from the oad:Query subclass if there is a specified query language. (But note that oad:KVPairQuerySelectors leave the choice of query language up to the consuming application.) If it is possible to process that language then continue, otherwise execute some abandonment procedure.
2. Extract the query from the object of has:Query.
3. If any of the Sources has rdf:type oad:AnySuchResource, then apply the extracted query to *all* datasets to which the query can be meaningfully applied; otherwise,
4. For each of the named Sources that is a dataset to which the query can be meaningfully applied, apply it to each but only to those.

For the queries below as examples we should then ask: is there a formalization of each query and a realistic implementation of the guidance, such that applying them together allows answers using the given ontologies?

Competency Question: To which resources, whether explicitly listed in the Target or not, should a consuming application apply the body of an annotation?

Competency Question (resource type specific): Given a specific resource that is serialized as an XML document, to which portion of the document, as specified by an XPath query, does the annotation apply?

Competency Question (Domain specific): Given a Specific Resource that is a data set that can be represented as DarwinCore, to which portions of the data set, as described by a set of values for DarwinCore data properties, does the annotation apply?

Competency Question (Domain specific): What are all the current annotations about a specimen of a North American ant for which a new taxonomic determination has been suggested since 2008 and acted upon?

Questions like these and several other competency questions, and some SPARQL representations of them, are provided in [File S1].

In summary, we extend OA in OAD with a set of Expectation classes modeling the annotator's expectation of how consumers may modify their data, an Evidence class modeling the annotator's evidence for the assertions that the annotator makes in the body of the annotation, a set of Query Selectors to select portions of structured data sets, AnySuchResource to model the application of Query selectors to arbitrary resources, and supporting object properties hasEvidence, hasExpectation, and hasQuery.

## Discussion

Powerful ontologies attempt to subscribe to the “Open World Assumption”: if assertions cannot be concluded to be true, then they must not be assumed false. By contrast, the “Closed World Assumption” is that anything not concluded to be true is assumed to be false. Like most RDF-based ontologies, AO and OA both promote the Open World Assumption. One technical consequence is that there may be issues of knowledge representation that can be resolved in multiple ways. In functioning knowledge organization systems, engineering considerations may favor choosing one or another resolution of such ambiguities. Below we mention some such issues and describe some of the impacts of choosing one solution over another. By its silence on some of the issues, AO faced more ambiguity than OA now does. Critically, in the natural science collection domain we are implementing actionable annotation applications against mutable data, which are mostly kept in relational databases (cf. [17], [44], [ and 45])—the epitome of closed world systems. (But see also [46]–[48].) For those applications we also have a number of requirements, which many RDF-based annotation applications might share. Among these are semantically based exploration of the content of annotations and semantically based notification—to interested parties—of the publication of annotations.

### Opaque objects

When annotations must be transported through a network, and distributed only to interested parties whose interests may change over time, it may be difficult to untangle the responsibilities of the transport mechanism from the responsibilities of the annotation. Opaque objects are an example of such an entanglement. In a simple case, an annotation might contain assertions carried as strings in data properties, where the string content of a data property is interpretable by a human reader but is opaque to machine reasoners acting in accord with an ontology. For example, an annotation asserting a new identification for a snail might carry Evidence in the form of a text string: “I can see the characteristic purple color of this species at the tips of the apertural side of the branching spines of this individual.” Such an assertion is easily interpreted by a malacologist but not by an ontological reasoner. In a much more extreme scenario, an annotation system might support redaction; it could allow the encryption of all or parts of the Body of the annotation but not the Target, but only selected potential recipients might hold the key needed to decrypt the assertions of the Body. As the Target of the annotation is expressed in a form that is not opaque to the annotation transport system (and any reasoners it may invoke), the annotation can be delivered to interested recipients with some of the assertions made in the Body remaining opaque to the annotation system. In that scenario, all network participants who had registered an interest in the Target would know that such an annotation has entered the network, and they could take steps to request that the annotator provide them with further information, which could comprise simply another annotation with some less sensitive information in the Body. This corresponds to a common scenario for species occurrence data in which the exact geolocation of endangered species is available only to accredited users, while public consumers are delivered a coarser geolocation, such as a county name. Indeed, one could even provide two Body parts, one with encrypted exact location and one with the coarse location. In our earlier XML-based annotation modeling we proposed an Opaque Object as a part of the payload of an annotation. In our early proposals for extensions to AO, we retained part of this concept in the data property with the proposed predicate *asText*. The asText property gave us a place to assert that part of an annotation is of type Evidence and then easily attach a textural representation of the Evidence to that object. As mentioned earlier, OA addresses this issue with the much more technically advantageous Content in RDF. This allows the treatment of a number of opaque and non-opaque data representations in a uniform way, including the use not only of plain text, but also of XML, encoded binary data such as encrypted material, or in-situ image data within annotations. Thus, for example, an image encoded in Base64 binary encoding could be carried with an annotation and offered as the Evidence for the assertions of the Body.

### Associations between annotation targets and bodies

**Figure 4:**
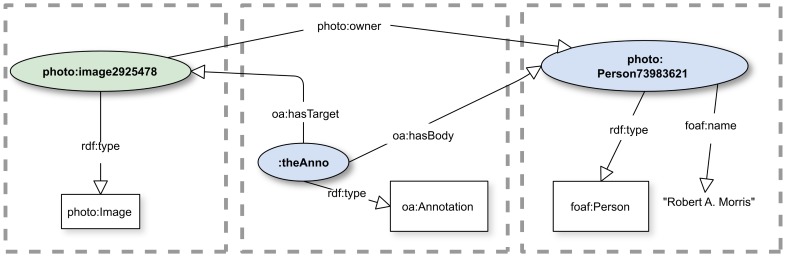
Linking components using only domain terms. The annotation uses an assertion within the imaginary photo vocabulary to associate an owner with the image that is the target. As described in the text, this approach cannot guarantee that the linkage arises from the annotation itself. See [RDF S3] for complete RDF representation.

The question arises: What are the consequences of making associations between a Target of an annotation and its Body? This issue is forced upon an annotator when there is a natural reason to make such an association using predicates from the domain vocabulary. For example, imagine an online photo gallery that provides machine APIs for provision of, and access to, metadata of images in the gallery along with unique identifiers for both images and contributors. (This is generally the case for such systems, e.g., [49], [50].) Suppose the specification for this API uses terminology whose prefix we denote with “photo:” and that it provides a predicate photo:owner. It is natural for an annotator to desire to provide an annotation whose Body asserts the identity of the owner of a particular image, and it is tempting to use photo:owner for this purpose, because the gallery service is presumed to understand such assertions. This would lead to an annotation such as illustrated in [Figure 4]. But this approach carries a surprising pitfall: it conveys nothing that allows the conclusion that this identification is in any way asserted by the Annotation. For example, it might have already been asserted by something having nothing to do with annotation at all. It is also not possible to tell whether attributes of photo:Person73983261 arise from the Target, the Body, or the Annotation. The heart of the issue is that, when an annotation asserts a domain object property that links a domain subject in the target with a domain object in the body, a competency question asking which triples were asserted in the body cannot be correctly answered. In addition, as we discuss later, the provenance of annotations and their components could be important for consumers of actionable annotations in making decisions about how to act on the annotation.

Thus, the boundary between knowledge provided by the Annotation and that provided by domain applications is not distinguishable in an annotation such as is depicted in [Figure 4]. Among the problems that can arise in this case is that, if a second annotation asserts a different owner, it could be difficult to decide how to resolve the result: are there two owners? is one of the annotations asserting that the other is in error? are the applications that produced the annotations even aware of the other annotation?

In general, annotations that assert a domain predicate that links a domain object in the Body to a domain object in the Target are fraught with the risk of misinterpretation. AO had no mechanisms for addressing this issue, but OA has several mechanisms that communities of practice could adopt, and, at the very least, make it possible to determine whether the association is coming from the annotation or not. We and others are exploring their utility. We discuss them next.

#### Associations using a proxy object

**Figure 5:**
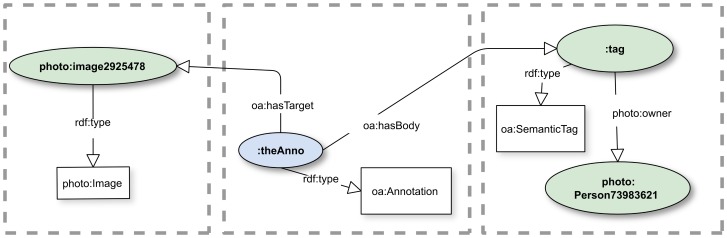
Linking using a SemanticTag. The photo:owner assertion is more tightly coupled to the annotation than in [Figure 4] to the extent that it is acting on an object whose type is a class on OA. The Open World Assumption notwithstanding, if the photo:owner predicate were removed, the foaf:Person would be unrelated to the annotation, and the tag would carry no knowledge. In this case, a consumer of the annotation could reasonably conclude that it received an incomplete annotation. See [RDF S4] for complete RDF.

Text-based tagging is a common use of annotation, and OA provides for it by declaring a class *oa:Tag* to which one can attach strings to Bodies using CNT in the manner described earlier. A subclass *oa:SemanticTag* of oa:Tag provides for the association of objects in arbitrary classes, typically from the domain providing the controlled vocabulary for the content of the Target and Body components of the annotation. That usage is illustrated in [Figure 5], wherein the predicate photo:owner is applied to an object that is *not* the image but with the intent that it applies to the Body object.

We must offer a caveat about the RDF language and its use. It is common, but often unnecessary, to build ontologies in which restrictions are placed on the class to which a given predicate can be applied. That class is called the *domain* of the predicate. The mechanisms for these restrictions are beyond the scope of this paper, but the effect for RDF is not to produce some sort of invalidity but rather to add the subject of the predicate—“:tag” in the Figure—to the predicate's domain. In the example, :tag would become a photo:Image object by inference. In the kind of systems that this simple example models, it would be usual, given an object such as :tag in the class photo:Image, that one would expect the service would then actually be able to deliver an image given only the identifier :tag. However, that would typically not be possible for something whose identifier was not issued by the gallery system itself. In short, *if* the putative image gallery specification placed a domain restriction on photo:owner, then ontological reasoners might produce some unintended consequences of this usage of oa:SemanticTag. Our own project avoids such pitfalls by avoiding unnecessary use of rdfs:domain in ontologies we design.

If, with the proxy solution, a consumer asserts owl:sameAs between the image and its proxy, queries on a semantic store risk return to the original problem of being unable to determine which assertions were made in the Body of the annotation. This solution is not immune to the Open World Assumption. It may impose a requirement on annotation systems for an immutable, i.e., Closed World, annotation document store, which allows recovery of the original annotation documents unaccompanied by assertions not made in them.

#### Encapsulation of the assertions in the body and in the target

RDF Named Graphs [51] provide a potential mechanism for encapsulating domain assertions in the target and the body such that it remains possible to determine which assertions were made in which. In such a scheme, the oa:Annotation would use the URI of the named graph given as the Target (resp. Body). This prevents associations of the sort illustrated in [Figure 4].

#### Associations using annotation properties in a community of practice

Another way to address the association problem is for applications or communities of practice to define properties of the annotation as expressing the association (without including an explicit assertion in the domain). This is the mechanism we use in FilteredPush applications. For example, consider the consumer of an annotation that asserts a dwcFP:Occurrence as the target, a dwcFP:Identification as the body, and an expectation of oad:Expectation_Insert. That consumer must understand that the annotation is implicitly requesting that the consumer create a new Identification and assert dwcFP:hasIdentification in the consumer's data store to link the new Identification to an existing Occurrence. As a practical matter, this means that software or humans responding to this Expectation must know how to translate the content of the dwcFP:Identification and associate it with the dwcFP:Occurrence, using whatever update mechanisms their database technology requires.

**Figure 6:**
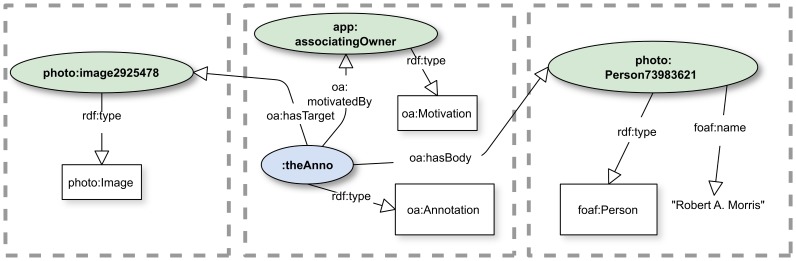
Strong association by use of oa:Motivation. By use of an oa:Motivation the annotator signals that the purpose of the annotation is to associate the owner with the image. See [RDF S5].

A community of practice could use oa:Motivation objects that correspond to common cases of association that may be required for their annotations. Such objects could then drive the business logic of consuming applications. Recall that an oa:Motivation is a skos:Concept. In the example at hand, we might define the skos:Concept “app:associatingOwner” and arrive at [Figure 6]. We specify the Motivation object with a prefix “app:” to indicate that it is not part of the API of the putative image service, but rather it is particular to the application (or community) dealing with annotation of images, which is not, per se, a concern of the gallery system.

### Handling multiplicity

If an annotation contains just one of each of Body, Target, Motivation, annotatedAt, Expectation, etc., then the semantics are clear. However, when there are multiples of any these, they are less so. An annotator could express several different things about a given Target at the same time or could express one thing about several Targets. Consider some cases. One case is when an annotator wishes to assert that each of ten records have the country name “Mongolia” misspelled as “Mongalia.” Another, more complex case is when an annotator wishes to assert that each of three records have both the country name “Mongolia” misspelled as “Mongalia” and have a misspelled collector's name, and wishes to assert evidence for the country name and evidence for the collector's name. Another case is when an annotator wishes to assert that one Target has a misspelled country name and another has a misspelled collector's name (giving evidence for each). The semantics of a chosen annotation model will determine which of these cases should be expressed as a single annotation and which as multiple annotations. Given the issues of relating Targets to Bodies with domain specific properties, it is not surprising that the semantics of OA suggest that multiplicity of assertions in a single annotation instance should be limited to the simplest possible cases, and that complex sets of assertions are best dealt with as multiple OA annotation instances.

To provide an example of multiplicity within the scope of OA, we discuss a multiplicity problem arising when a user wishes to express opinions about multiple errors in a putatively known database (in this case, three records in the Harvard Museum of Comparative Zoology (MCZ) delivered by the GBIF portal [36]). In this example, the user has no correction to offer, but is merely asserting that the data as delivered by GBIF originated at MCZ and are erroneous. This example arises from the email previously mentioned in the section **“Expectations for mutable data”** making those assertions in plain text and giving as evidence that the identifications assigned to the specimens represent species that are not found in the location where the specimens supposedly were collected. The email was sent by a herpetologist, Joseph Bernardo. We refer to this issue as the “Bernardo Assertions.”

An informal competency question may be stated for models of this situation, particularly as might be applied to an annotation store:

- Given an assertion, to what specimens is the assertion applied in some annotation?

There are several approaches to model the Bernardo Assertions, and each approach leads to a different SPARQL formalization of the informal competency question.

We model the targets of the Bernardo Assertions using the domain-specific dwcFP:DwCTripletSelector introduced in the section **“Selectors and queries.”** Of course, the Bernardo Assertions apply to *three* specimens, but, for each one, the Body, Evidence, and Expectation are identical. The current OA draft prohibits multiple Selectors on a single SpecificResource. It also requires exactly one oa:hasSource associated with a SpecificResource. Since Bernardo is making assertions about both GBIF and MCZ records, this requires six Targets, even though each record at MCZ will have the same DwC Triplet as its GBIF copy. If OA did not impose these particular uniqueness constraints, a smaller model could be constructed by placing three dwcFP:DwCTripletSelector objects on a single Target with two sources: the GBIF dataset *and* the MCZ dataset. This circumstance arises not because the data are mutable and the annotation is actionable—those give rise to the Expectation, which is meant to apply to all six records. Rather, the need to have a single Expectation and single Evidence applicable to all six records arises from the fact that there are data aggregators in the ecosystem. This is not an uncommon circumstance, which, in scientific data collections, gives rise to data quality control issues, e.g., [52]. A full model of the Bernardo Assertions is provided in [RDF S16].

The type oad:Expectation_Solve_With_More_Data signifies that the annotator expects that more data are needed to correct the error. It flags a problem with the data that the annotator can identify but not solve. In this case, examination by the collection management staff of the handwritten ledgers in which the specimens were originally cataloged into the MCZ resolved the issue identified by the annotator. It revealed that the same catalog numbers were improperly used for two sets of specimens. Subsequent digitization of these erroneously recorded the identifications from one set and locality data from the other set. In our model, this revelation could be captured in a response annotation, that is, one for which the Target was the original annotation and for which other details were adequate to allow an authorized person or software to make the necessary correction in the appropriate data set. This example shares many concepts with systems for software bug tracking and issue resolution. Indeed, we have been experimenting with augmenting quality control annotations with assertions from the Bug Ontology Model [53] together with the Marl ontology for characterizing subjective opinions [54]. Several complete examples are explored in [RDF S10, RDF S14, and RDF S15].

The current Community Draft of OA provides that, by default, each Body (if more than one) of an Annotation applies individually to *each* Target (if more than one) [55]. An earlier Community draft of OA prohibited multiple bodies [56], but we and others argued for more complex multiplicity use cases. Constructs for such cases, indeed, for *any* collection of components of an OA annotation, are provided in the current Community Draft. A solution was adopted that models several types of object containers, according as the contents of the containers should be regarded has having assertions apply to *all* of them independently of order (container type *oa:Composite*), to all of them *in order* (*oa:List*), or to exactly *one* of them (*oa:Choice*). These give applications that consume an annotation more guidance, and are of wider applicability, than the default behavior specified for Targets and Bodies.

The above considerations lead to an issue that remains an area of investigation for us. It may be seen by the fact that, while OA provides for default (and more complex) applicability of Body assertions to Targets, it disclaims them for provenance. That is, the provenance of the annotation is independent of the provenance of the parts of the annotation, e.g., the Body, as is exemplified in the introductory paragraphs of [57]. The general issue is that those wishing to extend OA, as we do with oad:Expectation and oad:Evidence, or, indeed even OA itself as it evolves by the addition of new classes, must always address the applicability semantics to best capture the usage of the new terms, especially as to advice to consuming applications. In essence, this issue is about requirements for extensions to OA. Presently, the only treatment of such requirements in OA is that for the extensions of the core oa:Motivations as treated in Appendix B of the OA Community Draft [56]. We currently treat multiplicity issues for oad:Expectation and oad:Evidence in the same way as OA does for Targets and Bodies.

Bernardo's information arose from the GBIF copy of the records, not the actual MCZ records. For a consumer to properly interpret Bernardo's assertions, more context for Bernardo's targeting, such as the date at which the putatively incorrect GBIF records were accessed, may be necessary. The reason for this is that GBIF is such a large aggregator that it re-indexes data only every few months, so it is quite possible that the error Bernardo reports could already have been corrected in the MCZ records. OA provides a predicate *oa:hasScope*, which references an object of arbitrary structure that can help a consuming application decide whether the resources it can examine correspond to those about which the Annotation is actually making assertions. We have not modeled Scope in the examples, but, if we did, the Targets would be the MCZ records, and the object of each oa:hasScope would be the corresponding GBIF record, given by the same selector but with the GBIF dataset as the object of its oa:hasSource. Alternatively, we could include the incorrect values as part of a query selector.

**Figure 7:**
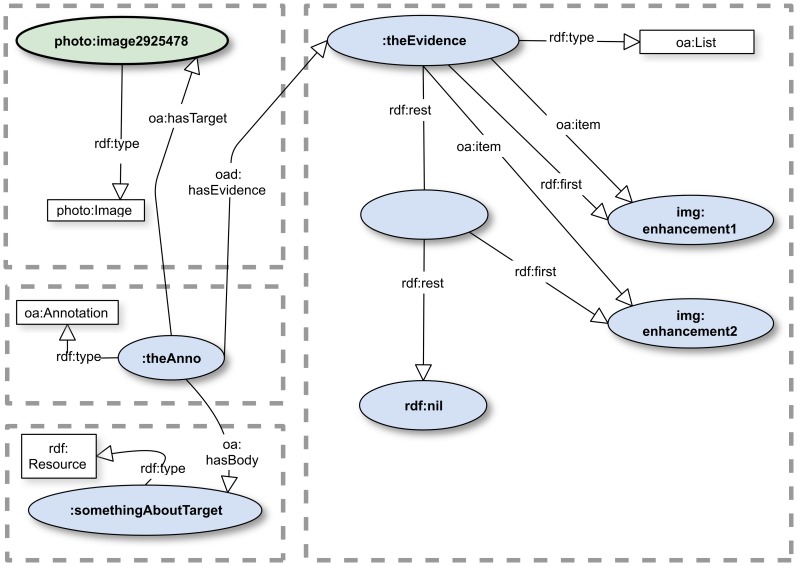
An ordered list of actions. The annotation models a suggestion that an ordered list of image enhancements be applied to the Target. See [RDF S6] for the complete RDF.

Finally, we illustrate the use of the oa:List multiplicity construct designed to signify that two objects must be processed in a particular order to process the annotation properly. We continue with our imaginary photo: domain and suppose that an annotator wishes to assert that some *enhanced* image provides evidence for the Body assertions, provided that the enhancement steps are accomplished in the correct order. Providing the provenance for image manipulation is a familiar problem in many sciences [58]–[60]. [Figure 7] shows the use of an oa:List to model the requirement that the Evidence items be evaluated in order by applications processing such an annotation. The example is truncated by assuming that the processing application understands that the enhancements are to apply to the Target image and that it is the *outcome* of the image processing workflow that provides the evidence. It is beyond the scope of this paper to delve into why there are blank objects in the model. The interested reader may consult Section 4.3 of [61] and Section 2.3 of [62].

It is tempting to believe that oa:List might provide a solution to the problem of multiple associations. For example, if an annotation has two Targets and two Bodies, one might declare a Body comprising an oa:List of two items and an oa:Target comprising a list of two items and assume that the Target/Body objects are associated pairwise. However, it is easy to see an immediate problem: to what would a third item apply if added to the list of Body items? In fact, oa:List is specified to mean nothing more than that the items in the list would result in a different outcome if processed in a different order from that of the oa:List. The Open World Assumption tells us that, if there is no explicit association of the objects in one oa:List with those in another, we gain nothing merely because the lists are the same size and the items call for natural associations.

An OA annotation forms a unitary whole. Each part of the annotation applies to all other parts. Absent external conventions in a community of practice, OA, unlike AO with its ao:AnnotationSet [63], provides no mechanism to allow an annotator to assert more than one thing at a time, such as one Target, two Bodies, and two Evidences, where one Evidence applies to one Body and the other Evidence to the other Body. In order to make two different assertions, an annotator must construct two annotation instances.

### Data provenance

There are a number of realistic reasons why the provenance of the putative MCZ records described in the Bernardo Assertions could become important, especially for a software system, such as our own, designed to process and distribute annotations. We mentioned above the scope issue arising from the possibility of a stale GBIF cache. oa:hasScope itself provides a kind of provenance, which arises commonly in heterogeneous distributed data environments in which data aggregators and forwarders are not always able to carry the full original scope of the records they serve, and complex machine-based workflows may act on data from multiple sources [64]–[66]. A distinction sometimes made in the workflow community is between *retrospective* and *prospective* provenance. The former is quite common now and results from scientific workflow systems recording how a particular workflow was executed and how final and intermediate data products were derived. By querying such provenance information, data lineage and other data dependency information can be obtained. The latter “captures an abstract workflow specification as a recipe for future data derivation” [67] p. 447, i.e., the workflow itself is a form of provenance, able to explain to some extent how one arrives at a certain result. This dichotomy fits well with a system, such as FilteredPush, which distributes annotations to data providers so that they may correct the underlying data. On the one hand, Kepler workflows in a FilteredPush node can launch annotations about curation events identified in the Kepler Kurator package [68] and record the corresponding (retrospective) provenance information in the annotation. On the other hand, when an annotation is launched into a FilteredPush network, a triage component of the network can prospectively invoke workflows to assist in determining the fate of the annotation in the network. Note that the annotation illustrated in [Figure 7] offers a kind of prospective provenance.

A key provenance issue for annotation of data again arises from the difference between marginalia on paper and the annotation of data. An annotator of a data set may have some expectation that the data curator will change her data, and the data curator may very well need provenance information in order to decide whether to accept or reject the proposed change. This is analogous to the situation mentioned earlier in classical copy editing, but in that case the author is likely to already know the origins of the proofreaders' marks. The main point is that the importance of annotation provenance is highly dependent on context.

### Annotation conversations

If an annotation B has Target an annotation A, we say that B is a *response* to A. By “annotation conversation” we mean a collection of annotations that forms a directed graph under the relation oa:hasTarget and is connected when considered as an undirected graph. Annotation conversations about mutable data can provide a history of proposed, implemented, or rejected changes to a resource in much the same way as change tracking systems do in document processing systems or bug tracking systems do for software management. See [RDF S10] for an extended example.

The Open World Assumption provides the possibility that an annotation conversation is not an acyclic graph, because even if A does not yet exist at the time B is created, nothing prevents B from asserting *its* target is the URI that may ultimately be assigned to A. In that case, B is a response to A and A is a response to B. That said, simple acyclic conversations are important in a network of providers of actionable annotations, because they allow, among other things, for recipients to signal interested parties how they acted on the Expectation expressed in an annotation. The Expectation oad:Solve_With_More_Data also might provoke a conversation, perhaps of slightly greater complexity, in case a response in fact provides more data as further Evidence for the original annotation. These two important cases usually generate no cycles, but annotation processing software may have to be prepared to handle cycles gracefully. Cyclicity is not restricted to oa:hasTarget. Any RDF predicate can fall afoul of it for the same reason that oa:hasTarget can. However, SPARQL provides a mechanism for dealing with property chains without running afoul of cyclicity [69], and this mechanism can be applied by annotation consuming software that can make SPARQL queries. The details are beyond the scope of this paper. Such queries could formalize, and answer, a competency question such as:

- What are all the current annotations of a specimen record that assert collection latitude greater than 90 degrees and for which there is presently no response indicating that a correction has been made?

That question has a SPARQL formulation in [File S1].

### Annotation provenance, persistent annotation document stores, and serialization

Earlier we briefly mentioned that the provenance of an annotation, as opposed to that of data it treats, is important for actionable annotations, because this provenance can help the consumer decide whether to act in the manner the annotation suggests. This kind of provenance is difficult to provide entirely in an RDF environment, because it is a delicate matter to detect what has been added to an RDF object after its creation. (That circumstance arose in the discussion of [Figure 4].) The consequence is that annotation systems requiring annotation provenance must both adopt some kind of persistent immutable annotation store and also provide for the Open World knowledge extensibility mechanisms available through RDF, including support for ontology-based machine reasoning where desired. In addition, if the underlying annotated objects are not themselves in some kind of store providing at least the possibility of object provenance, then supporting this must also be considered because such provenance also can assist the decision whether or not to act on an annotation. Although the details are outside the scope of this paper, our characterizations of Annotation systems by how annotations are stored and distributed can impact the engineering choices for this issue more than do the knowledge representation details. Furthermore, the underlying FilteredPush architecture can support each of the four kinds of annotation systems we described, so we mention only briefly some strategies adopted in our current implementations and in several systems of others who have adopted OA. For the purposes of this paper, the important issue is that each of these strategies supports basic annotation provenance. For example, the persistent store for the Scholarly Editions annotation platform [70] uses Named Graphs for annotations and to support SPARQL queries, but uses the web itself as its “data” store since Scholarly Editions principally annotates documents. We considered the use of Named Graphs for FilteredPush, but, particularly in the face of possibly huge numbers of software-generated annotations, we presently use the Fedora Commons [71] document store for a persistent annotation store and a separate triple store managed by Apache Jena [72] to support reasoning and SPARQL queries. For efficiency, our data store also includes the popular NoSQL database MongoDB [73], populated as needed. The AnnoSys project [74] also based on OA for annotation of natural science specimen data, uses Selectors based on XPath queries that are against a single persistent annotation document store based entirely on XML. Unlike FilteredPush and the Scholarly Editions system, AnnoSys must hard code any RDF inferencing. In FilteredPush deployments, we generate annotations with tools of Apache Jena, driven by configurations that depend on the annotation ontologies and the domain ontology, in our case dwcFP. Annotations are serialized as RDF/XML for launch into the network, but Jena can easily serialize in more web-friendly formats, including JSON-LD [75]. The latter is indeed what OA recommends, but we presently do not use it since our web presentations are generally handled by lightweight additions to the specimen management systems themselves, even when those are web-based. The FilteredPush architecture and its various deployment implementations are beyond the scope of this paper, but a typical architecture is illustrated in [Diagram S1].

### Rules, filters, and validation

As illustrated above, OA offers several ways to address certain issues arising from its high level of generality and attention to the Open World Assumption. In a practical system, we find that settling on a single way for use within a network helps us avoid, or at least detect, ambiguities when annotations are in motion or need to be integrated across multiple annotation stores.

In the networks we are building, participating nodes may have one or more roles as producers or consumers of data and as producers or consumers of annotations. In such a mix of roles, it is particularly important to assure that the nodes behave in semantically consistent ways. Rather than requiring that producing and consuming applications build the rules for this cooperation into their business logic code, we instead provide rule configuration files that can be examined at runtime. Our rules are designed to detect *violations* of the collaborative principles, so that collaborators can ignore annotations containing such violations. We do this because the Open World Assumption allows that an annotation could have assertions that are both true and false. Testing for truth will not, per se, also test for falsehood. Consider the earlier examples describing the dwcFP:DwCTripletSelector and consider the reasonable rule that there must be exactly one each of dwc:institutionCode, dwc:collectionCode, and dwc:catalogNumber in a valid data record specified in the Target of those examples.

One could imagine defining classes of Selectors using OWL cardinality restrictions [27] p. 257. This usage could be expressed as:
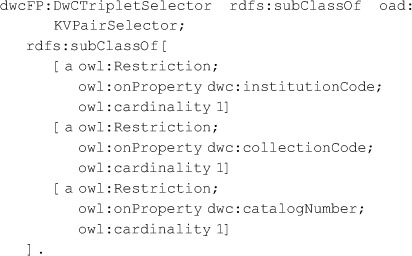



Appealing as this might seem, “Darwin Core Triplet” unfortunately has no formal definition in the Darwin Core specification [76]. Furthermore, several variants of it are in use. Some usage omits the institutionCode where the collectionCode is given by a widely used acronym that identifies an institution that has only one collection. Some use the Darwin core terms institutionID and collectionID rather than institutionCode and collectionCode. Consequently, in the interest of greater applicability, our dwcFP ontology does not itself specify these constraints. This is not debilitating, because many OWL reasoners can apply rules of this form declared in a rule set accessed at run time separately from the ontologies in use. With only a slight increase in the complexity of the owl:Restrictions, we could cover the most commonly used triplet structures. However, with such declarations of the restrictions, we would find, as in the case with specifying rdfs:domain on properties, that the Open World Assumption brings surprises. For example, if an Annotation were specified using a DwCTripletSelector that specified only the catalogNumber, an OWL reasoner might only be able deduce that there is a logical contradiction in the store holding this and other Annotations. But other assertions, not a rule violating specifier in that store, might be the source of that contradiction. In addition, some standard subsets of OWL2 do not permit such cardinality restrictions, which could limit use of some of the varieties of tractable ontology design that OWL2 supports.

Instead, we have chosen to express rules in FilteredPush deployments using SPARQL queries. The SPARQL query below will return all, and only, those annotations that specify a Selector that violates the prohibition on targeting ill-formed triplets.
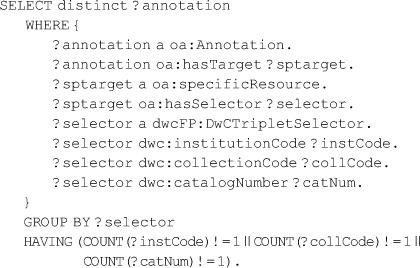



Note that this query returns all the *annotations* whose DwCTripletSelector is ill-formed. The rule is about the validity of the annotations, not of the data.

This approach brings several immediate advantages. First, there may be annotations whose Selector does not meet the rule but nevertheless carry useful information. Such “invalid” annotations might be fetched from an annotation knowledge base without problem if the application is taking no action that is affected by those associations (e.g., the cases of missing institutionCode mentioned above). Second, query-based filtering maps well into semantic Publication/Subscription (Pub/Sub) models, which frequently represent subscriptions as queries [77]–[79]. In turn, human and software agents at the network periphery, or within the network, can register an interest (as a query) that serves as a filter against which the Pub/Sub subsystem can notify those agents of new knowledge published to the network in the form of annotations and the actions taken based upon them.

SPARQL and OWL rule representation notwithstanding, it is important to keep in mind that RDF is descriptive, not proscriptive. A consequence is that systems implementing actions upon actionable annotations will generally need some agreements between annotation producers and consumers that cannot be expressed in RDF technologies. For example, such systems might require explicit agreement that processing an oa:List will in fact take place in order, or that an oad:KVPairQuerySelector be interpreted as a set of criteria linked by “and” when composed into a native query language.

### Evaluation

There is no widely agreed upon framework for evaluating ontologies, but a community-based proposal characterizing the quality issues for the entire ontology life cycle has recently emerged [80]. That proposal rests on five criteria, which are not entirely independent, at least as to evaluation and the role of competency questions:

1. Can humans understand the ontology correctly? *(Intelligibility)*
2. Does the ontology accurately represent its domain? *(Fidelity)*
3. Is the ontology well-built and are design decisions followed consistently? *(Craftsmanship)*
4. Does the representation of the domain fit the requirements for its intended use? *(Fitness)*
5. Does the deployed ontology meet the requirements of the information system of which it is part? *(Deployability)*

It's quite difficult to make quantitative measures of these criteria, and the principal ones explored in the proposal surround ontologies built for applications on corpora of natural language, to which we rarely apply our OAD extensions. Thus, while we have not yet attempted quantitative measures, we do address all five criteria explicitly in a qualitative fashion:

1. (Intelligibility): Vocabulary terms are given human readable names (e.g. oad:hasEvidence) following typical OWL naming conventions. We make liberal use of comment properties to document terms in oad.rdf and our domain-specific ontology dwcFP.owl. The latter plays an important role in Deployability evaluation. In addition, we have a library of handcrafted illustrative annotations designed for human readability (including the choice of serialization, N3) and as models for machine generation of data annotations.
2. (Fidelity): We regard the domain as the actionable annotation of queryable, mutable data. Our introduction of QuerySelectors, along with the Evidence class, allows us to treat such data for structured, semi-structured, or unstructured data, provided only that the QuerySelectors can model the domain of the data itself. Evaluating whether that criterion is met in turn hinges on the success of deployments.
3. (Craftsmanship): We consistently adhere to two design principles: (a) Separate the concerns of transport, annotation, and the domain, placing only concerns of annotation within the annotation ontology; (b) Avoid over specification (e.g., un-necessary declarations of rdfs:domains.)
4. (Fitness): Our principal fitness evaluation is based on competency questions expressed as SPARQL. We test those rules against example instance documents, both hand-crafted and machine-generated. In addition, we validate a number of SPARQL-based rules that simplify deployability without reducing Fidelity. Evaluation of Fitness and Deployability may be difficult to separate.
5. (Deployability): We have built and are testing several instances of networks of data annotation producers and consumers, as well as a standalone producer with a restful web service API implemented in Java. In turn, our network designs require that the ontologies be adequate for the provision of notification of annotations, filtered according to the scientific and curatorial interests of the recipient. Critically, the filtering needs of the data curators whose data are the target of an annotation must be expressible by the mechanisms of the deployment. Use of a notification infrastructure that includes SPARQL queries on a triple store accomplishes this with no further vocabulary than OA, OAD, and a data domain ontology.

### Conclusions

Semantic annotation of data at and below the record level shares much in common with document annotation, but a small number of additional concepts are needed in practical applications, such as data quality control and provision of missing data either by human experts or software agents. Central among these are concepts that allow the annotating agent to provide evidence that supports the corrections and additions and concepts with which the annotators can indicate what action they expect the original data holders to take. OA provides a useful separation between what is annotated and what is asserted about it. Because these assertions can be scientific propositions, we needed to add the ability to model evidence for them using domain vocabularies. Because annotated data may be mutable, we needed to add vocabulary to express the annotator's opinion about how the annotated data should be changed. Adding these models at the top level of an annotation makes it possible, as an annotation is processed moving through a software system or network, to separate them, extract or add information to them independently, and reassemble them into the same or a derived annotation. Representation of annotations supporting the Open World Assumption can give rise to an engineering requirement to deploy a document store to provide provenance for original annotation documents.

In order to keep annotation ontologies general and flexible, particularly when annotations on distributed data are themselves being distributed, annotation systems should tolerate, but uniformly control, ambiguities arising from Open World Assumptions in the knowledge representation. One approach is to use query-based rules that select only unambiguous annotations to be used in any resulting data changes, while at the same time allowing for less strict annotations to coexist and be used where particular ambiguities do not render the annotation unfit for every possible knowledge representation purpose. Treating queries themselves as semantically significant objects in a query language appropriate to the data storage can model assertions that all data returned by specific queries should have particular properties. This can make it easy for annotation consumers to insert or correct particular data semi-automatically, and for annotators to construct annotations in a form consistent with consumer needs. In the current draft of OA, provision of the scope of applicability to queries requires the use of Selectors that refer to a specific dataset (its oa:Source). However, it is possible to define domain specific classes of datasets appropriate to a particular query language, along with a specially defined unresolvable class for which, if the Source is typed to that Class, a rule can dictate that the annotation is applicable to any other dataset or record to which the query can be applied, whether or not extant at the time of the annotation.

## Supporting Information

Diagram S1 **FilteredPush Deployment.** Partial architecture of an actual FilteredPush specimen metadata annotation network.
(TIF)

File S1 **SPARQLcompetency questions.** A set of SPARQL-based competency questions for OA and OAD. These have been tested with a Jena Fuseki SPARQL endpoint, including reasoning over the OA, OAD, and dwcFP owl ontologies. A sample working endpoint is documented in the file.
(TXT)

Ontology S1 **Data annotation ontology extensions to the Open Annotation Ontology.**
(RDF)

Ontology S2 **OWL ontology modeling the DarwinCore vocabulary.**
(OWL)

RDF S1 **RDF for** **Figure 2** **.** Complete annotation in RDF N3 notation, corresponding to [Figure 2]. Provides an example that asserts a new taxonomic identification on a specimen with URI huh:A-barcode-00107800. The annotator expects that a consumer with adequate authority will accept the assertion that this is the appropriate taxonomic identity and will update the consumer's records.
(N3) Click here for additional data file.

RDF S2 **RDF for** **Figure 3** **.** Complete annotation corresponding to [Figure 3]. Similar to [RDF S1], but illustrates use of the dwcFP:DwCTripletSelector assuming that the annotated specimen record does not have its own URI.
(N3) Click here for additional data file.

RDF S3 **RDF for** **Figure 4** **.** Complete annotation corresponding to [Figure 4], illustrating use of domain links to detail the relation between Target and Body of an annotation. The example is based on an imaginary photo gallery service that can associate a person with a photo using a predicate photo:owner and can generate annotations with a service having a URI photo:annotationGenerator. The main text explains why this model, which asserts a direct link between the image and the owner, is counter-productive.
(N3) Click here for additional data file.

RDF S4 **RDF for** **Figure 5** **.** Complete annotation corresponding to [Figure 5]. Similar to [RDF S3] but illustrates the use of OA semantic tagging to make the association.
(N3) Click here for additional data file.

RDF S5 **RDF for** **Figure 6** **.** Complete annotation corresponding to [Figure 6]. Similar to [RDF S3], but illustrates the use of an OA:Motivation to provide guidance to consuming applications.
(N3) Click here for additional data file.

RDF S6 **RDF for** **Figure 7** **.** Complete annotation corresponding to [Figure 7]. Synthetic example that offers an enhanced image as Evidence for Body assertions. Illustrates oa:List to model that the enhancements must be done in order.
(N3) Click here for additional data file.

RDF S7 **Use of an image of a botanical sheet.** Realistic complete annotation that illustrates the provision of oad:Evidence using an oa:FragmentSelector to circumscribe part of the image of a botanical sheet. That part contains the taxon name that the annotator offers in the Body, and is thereby given as evidence that the name should apply to the specimen on the sheet. The annotation provides two Evidence objects, one fully structured and the other human-centric.
(N3) Click here for additional data file.

RDF S8 **Annotation evidence based on image of a morphological character.** Example provides an annotation containing a new taxonomic determination based on a morphological character observed in a region of interest (roi) in an image of a specimen. The example exhibits the use of an oa:SvgSelector to specify the roi.
(N3) Click here for additional data file.

RDF S9 **Annotation with image processing evidence.** Realistic complete example similar to [RDF S7] but asserting in more detail that the evidence is the result of OCR applied to the region of interest. In particular, the region must be first selected, and the OCR applied to that, not to the entire image. This is a more extensive example than [RDF S8].
(N3) Click here for additional data file.

RDF S10 **Extended response annotation.** Realistic example that illustrates a response to [RDF S8], which provides evidence for a taxonomic determination, but of a sort insufficient for the responder on policy grounds. The annotation indicates that the evidence is convincing, but does not meet local policy. The example uses the Bug Ontology Model and the Marl opinion ontology to model the disposition of the annotation.
(N3) Click here for additional data file.

RDF S11 **Annotation commenting on a georeferencing error.** One of three annotations making assertions of georeferencing errors. These three files participate in one of the SPARQL Competency Questions that asks for annotations that assert an out-of-range latitude, but for which no response annotation has been given indicating that the data have been corrected. The full SPARQL is given in the file CompetencyQuestions.txt [File S1]. See also [RDF S12] and [RDF S13].
(N3) Click here for additional data file.

RDF S12 **Second annotation commenting on a georeferencing error.** Similar to [RDF S11].
(N3) Click here for additional data file.

RDF S13 **Third annotation commenting on a georeferencing error.** Similar to [RDF S11].
(N3) Click here for additional data file.

RDF S14 **Annotation correcting an error.** This is a response annotation asserting that the error in [RDF S11] has been fixed.
(N3) Click here for additional data file.

RDF S15 **Annotation declining to correct an error.** This is a response annotation noting that [RDF S12] correctly suggests an error, but the annotator is declining to fix it for lack of evidence provided in the original. Note also that in the example set there is no annotation at all responding to [RDF S13], so the aforementioned Competency Question should return both [RDF S12] and [RDF S13].
(N3) Click here for additional data file.

RDF S16 **Bernardo Assertions.** Complete annotation implementing the “Bernardo Assertions” described in the text.
(N3) Click here for additional data file.

Table S1 **Table of Prefixes.** Vocabulary prefixes and namespaces used in the paper.
(PDF)

Table S2 **Glossary of Acronyms.**
(PDF)
