# Supplementary figures and images for: Semantic Annotation of Mutable Data
Source: PLoS One. 2013 Nov 4;8(11):e76093. doi: 10.1371/journal.pone.0076093 (PMC3817185; doi:10.1371/journal.pone.0076093)

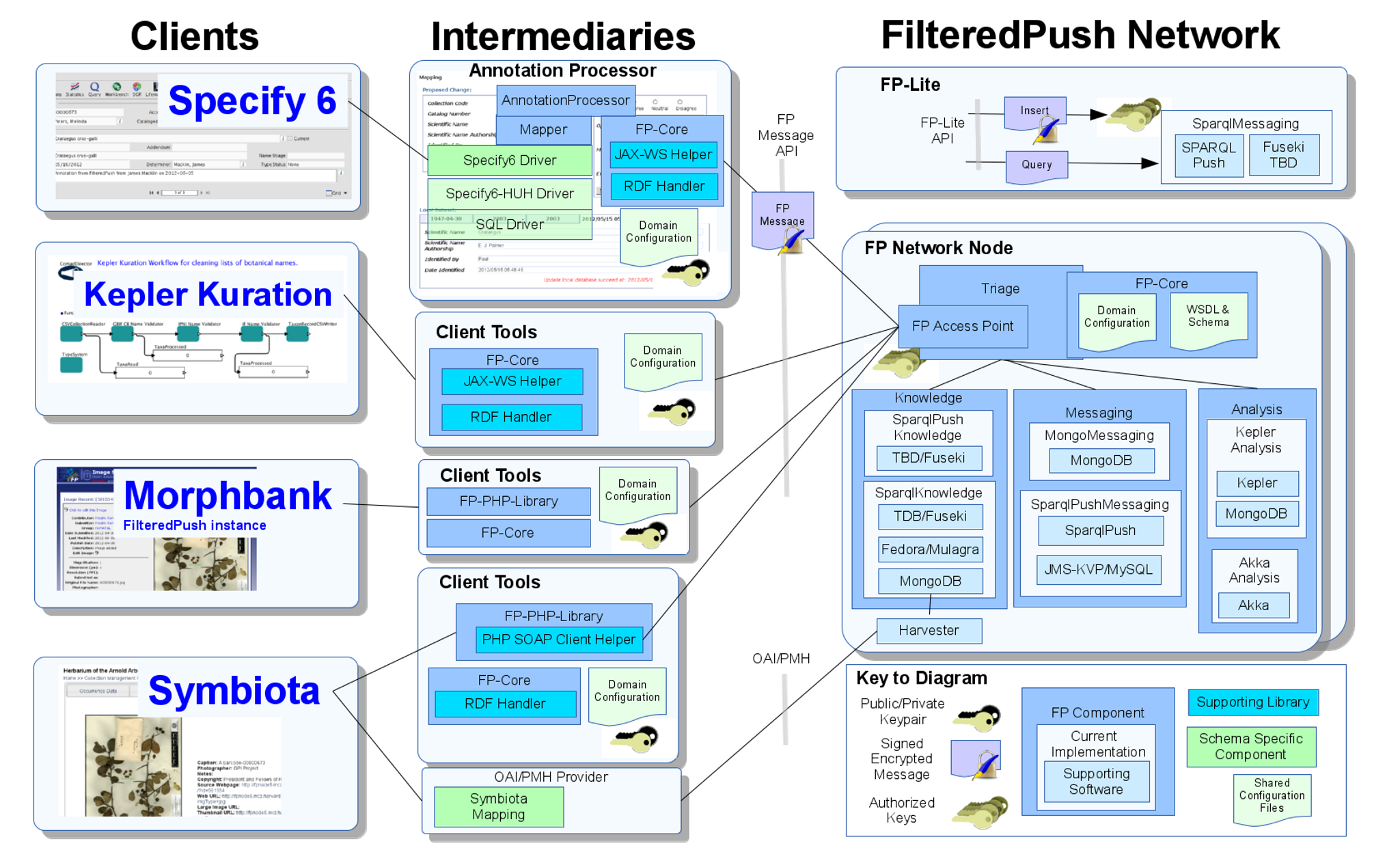

Supplement: Diagram S1 — FilteredPush Deployment. Partial architecture of an actual FilteredPush specimen metadata annotation network. (TIF) [file pone.0076093.s001.tif]
