# Supplementary material for: Semantic Annotation of Mutable Data
Source: PLoS One. 2013 Nov 4;8(11):e76093. doi: 10.1371/journal.pone.0076093 (PMC3817185; doi:10.1371/journal.pone.0076093)
Supplement: Table S1 — Table of Prefixes. Vocabulary prefixes and namespaces used in the paper. (PDF) [file pone.0076093.s021.pdf]

Prefixes		
**Prefix**	**Vocabulary**	**Namespace**
:	Default prefix	Depends on application
app:	Imaginary application built against the photo: gallery service	http://filteredpush.org/ontologies/examples/app/
cnt:	Representing Content in RDF	http://www.w3.org/2011/content#
dwc:	TDWG Darwin Core	http://rs.tdwg.org/dwc/
dwcFP:	FilteredPush Darwin Core OWL model	http://filteredpush.org/ontologies/oa/dwcFP.owl#
foaf:	FilteredPush Darwin Core OWL model	http://xmlns.com/foaf/0.1/
gbif:	GBIF data portal	http://data.gbif.org/datasets/
huh:	Shorthand for the set of Herbaria (A, GH, FH, ECON, AMES, NEBG)	http://purl.oclc.org/net/edu.harvard.huh/guid/uuid/
image:	Imaginary terminology for describing digital image processing operations	http://filteredpush.org/ontologies/examples/image/
oa:	Open Annotation Ontology	http://www.w3.org/ns/oa#
oad:	Proposed extensions to OA to support mutable data	http://filteredpush.org/ontologies/oa/oad#
owl:	OWL ontology language	http://www.w3.org/2002/07/owl#
photo:	Vocabulary for services by an imaginary image gallery service	http://filteredpush.org/ontologies/examples/photo/
rdf:	Resource Description Framework	http://www.w3.org/1999/02/22-rdf-syntax-ns#
skos:	W3C Simple Knowledge Organization System	http://www.w3.org/2004/02/skos/core#
