# Supplementary material for: Semantic Annotation of Mutable Data
Source: PLoS One. 2013 Nov 4;8(11):e76093. doi: 10.1371/journal.pone.0076093 (PMC3817185; doi:10.1371/journal.pone.0076093)
Supplement: Table S2 — Glossary of Acronyms. (PDF) [file pone.0076093.s022.pdf]

Glossary of Acronyms	
**Acronym**	**Meaning**
A	Herbarium of the Arnold Arboretum, one of the Harvard University Herbaria.
AO	Annotation Ontology
CNT	Content in RDF specification
DwC	Darwin Core
GBIF	Global Biodiversity Information Facility
GO	Gene Ontology
HAO	Hymenoptera Anatomy Ontology
MCZ	Museum of Comparative Zoology, Harvard University
OA	Open Annotation (Ontology)
OAC	Open Annotation Consortium
OAD	Open Annotation Ontology for Data
OBO	Open Biomedical Ontologies
OWL	Web Ontology Language
RDF	Resource Description Framework
RDFS	RDF-Schema
SKOS	Simple Knowledge Organization System
SPARQL	(Name of the RDF Query Language)
SQL	Structured Query Language
TDWG	Biodiversity Informatics Standards (TDWG), formerly “Taxonomic Databases Working Group”
URI	Uniform Resource Identifier
XML	eXtensible Markup Language
